# Investigating the early impacts of the COVID-19 pandemic on modifiable risk factors for cancer and chronic disease: a repeated cross-sectional study in Alberta, Canada

**DOI:** 10.17269/s41997-022-00685-x

**Published:** 2022-09-01

**Authors:** Geneviève Jessiman-Perreault, Alvin Li, Nicole Frenette, Lisa Allen Scott

**Affiliations:** 1 Cancer Prevention and Screening Innovation, Provincial Population and Public Health, Alberta Health Services, Calgary, AB Canada; 2 Department of Public Health Sciences, Dalla Lana School of Public Health, University of Toronto, Toronto, ON Canada; 3 Department of Oncology, Cumming School of Medicine, University of Calgary, Calgary, AB Canada; 4 Department of Community Health Sciences, Cumming School of Medicine, University of Calgary, Calgary, AB Canada

**Keywords:** COVID-19, Tobacco use, Healthy diet, Alcohol use, Physical activity, Socioeconomic factors, Risk factors, COVID-19, usage de tabac, régime alimentaire sain, consommation d’alcool, exercice physique, facteurs socioéconomiques, facteurs de risque

## Abstract

**Objectives:**

This study contributes to empirical evidence by examining the impact of the first and second waves of the COVID-19 pandemic on modifiable risk factors (MRF) and whether these patterns differ according to level of material deprivation among people living in Alberta.

**Methods:**

Using data from a repeated cross-sectional provincial health survey (Alberta Community Health Survey (ACHS): 2018–2021), we conducted logistic regression analyses examining the impacts of the COVID-19 pandemic on meeting national guidelines on four MRFs (tobacco use, physical activity, fruit and vegetable consumption, alcohol use) (*n*=11,249). We compared population-level changes in MRFs from one year before the COVID-19 pandemic (March 2019–February 2020) to one year during the pandemic (March 2020–February 2021) in Alberta. We also assessed whether these trends differed by a measure of material deprivation.

**Results:**

Compared to the pre-COVID-19 period, the fully adjusted odds of meeting recommended guidelines for fruit and vegetable consumption (OR=0.42) decreased during the pandemic. Individuals experiencing high material deprivation had lower odds of meeting recommended guidelines for physical activity (OR=0.65) and higher odds of not being current tobacco users (OR=1.36) during the pandemic versus during the pre-pandemic period.

**Conclusion:**

At a population level, analyses from the ACHS showed minimal impacts of the first year of the COVID-19 pandemic on MRFs, besides fruit and vegetable consumption. Yet, stratifying results showed statistically significant differences in pandemic impacts on MRFs by level of material deprivation. Therefore, understanding the influence of material deprivation on MRFs during the pandemic is key to tailoring future public health interventions promoting health and preventing cancer and chronic disease.

## Résumé

**Objectifs:**

Notre étude contribue aux preuves empiriques en examinant l’impact de la première et de la deuxième vague de la pandémie de COVID-19 sur les facteurs de risque modifiables (FRM) et en déterminant si ces tendances diffèrent selon le niveau de défavorisation matérielle chez les personnes vivant en Alberta.

**Méthode:**

À l’aide des données d’une enquête de santé transversale provinciale répétée (Alberta Community Health Survey [ACHS] : 2018-2021), nous avons mené des analyses de régression logistique pour étudier les impacts de la pandémie de COVID-19 sur le respect des directives nationales pour quatre FRM (tabagisme, activité physique, consommation de fruits et légumes, consommation d’alcool) (*n* = 11 249). Nous avons comparé les changements populationnels dans les FRM une année avant la pandémie de COVID-19 (mars 2019 à février 2020) et une année durant la pandémie (mars 2020 à février 2021) en Alberta. Nous avons aussi cherché à déterminer si ces tendances différaient selon un indicateur de défavorisation matérielle.

**Résultats:**

Comparativement à la période ayant précédé la COVID-19, le rapport de cotes entièrement ajusté pour le respect des directives de consommation de fruits et légumes recommandées (RC = 0,42) a diminué durant la pandémie. Les personnes aux prises avec une importante défavorisation matérielle ont présenté une probabilité plus faible de respecter les directives recommandées pour l’activité physique (RC = 0,65) et une probabilité accrue de ne pas être des consommateurs actuels de produits du tabac (OR = 1,36) durant la pandémie qu’avant la pandémie.

**Conclusion:**

Pour l’ensemble de la population, nos analyses de l’ACHS montrent que la première année de la pandémie de COVID-19 a eu très peu d’impact sur les FRM, sauf pour la consommation de fruits et légumes. Pourtant, la stratification des résultats montre des écarts significatifs dans les impacts de la pandémie sur les FRM selon le niveau de défavorisation matérielle. Il est donc essentiel de comprendre l’influence de la défavorisation matérielle sur les FRM durant la pandémie pour adapter les futures interventions de promotion de la santé et de prévention du cancer et des maladies chroniques.

## Introduction

The potential long-term impacts of COVID-19 pandemic-related public health restrictions on individual health behaviours, including those that are known to increase some people’s risk of developing cancer and other chronic conditions, remain largely unknown (Arora & Grey, 2020; Zajacova et al., 2020) and emerging results indicate inconsistent impacts of the pandemic on the modifiable risk factors (MRF) for chronic diseases. Therefore, individual health behaviours such as tobacco use, physical activity, fruit and vegetable consumption, and alcohol use warrant further consideration due to their association with cancer and chronic disease risk (Beaglehole et al., 2011). In Canada, almost 40% of cancer cases are attributed to 16 MRFs (Pader et al., 2021). Tobacco use, lack of physical activity, low fruit and vegetable consumption, and alcohol use were among the top MRFs attributable to cancer incidence in Canada (Poirier et al., 2019). These MRFs have also been found to be associated with risk of chronic disease in Ontario (Ng et al., 2020). Research has shown that meeting recommended guidelines for these MRFs can have a profound impact on reducing cancer incidence in Canada by 2047 (Poirier et al., 2019).

**Fig. 1:**
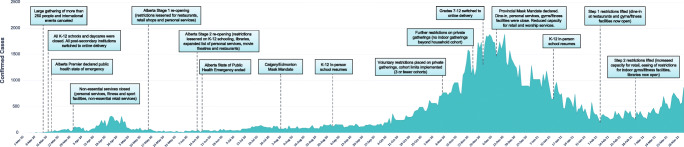
Timeline of COVID-19 public health restrictions in Alberta, March 2020–February 2021. Figure adapted from Centre for Health Informatics. (2021). Alberta’s Pandemic Response. Retrieved from https://covid-tracker.chi-csm.ca/

In Alberta, the setting of the present study, public health measures to curtail the spread of COVID-19 (i.e., gathering size limits; closures of non-essential businesses such as gyms, restaurants, and bars; mask mandates; and restrictions on non-household gatherings) were implemented and informed by changing case counts and hospitalizations. Figure 1 provides a timeline of public health restrictions implemented in Alberta from March 2020 to February 2021. Alberta, along with Ontario and BC, were among the first Canadian provinces to declare a state of emergency (Breton & Tabbara, 2020). Alberta quickly increased stringency of restrictions during the first wave but was one of the first provinces to remove restrictions at the tail end of the first wave and slowly increased stringency of measures leading into the second wave (Cameron-Blake et al., 2021). This resulted in Alberta’s approach being deemed “pro-business” (Cameron-Blake et al., 2021, p.11) as some non-essential businesses (e.g., bars, restaurants, and gyms) stayed open for longer than in other provinces. These differences in provincial approaches to COVID-19 pandemic restriction may impact some MRFs such as physical activity and alcohol use. Therefore, differences in public health measures by region may impact the relationship between the pandemic and MRFs.

Additionally, the consequences of the COVID-19 pandemic on individual health behaviours are expected to vary based on social determinants of health due to their influence on where people live, work, and play, and therefore contribute to inequities in health outcomes. The COVID-19 pandemic has also exacerbated existing inequities, in that those already part of disadvantaged groups are expected to experience the greatest impacts (Zajacova et al., 2020). This is particularly significant given that research has previously established that vulnerable populations are associated with having poorer health behaviour practices in general (Mudryj et al., 2019), as well as experiencing disproportionate impacts of COVID-19 (Zajacova et al., 2020). Thus, the impact of the COVID-19 pandemic on health behaviours has the potential to widen existing health inequities.

Overall, the COVID-19 pandemic has profoundly changed the context in which population and public health professionals work to intervene. It is essential to understand how people’s health behaviours changed during the COVID-19 pandemic, and the related sociodemographic characteristics of such changes, to design targeted interventions to improve health outcomes and minimize inequities or unintended harms. Our team of implementation scientists within Alberta Health Services develops and tests both population-level and targeted innovations to reduce cancer and chronic disease risk. To be responsive to the emerging/changing nature of MRFs in Alberta, we need to better understand how MRFs are changing during the COVID-19 pandemic, in which groups and why. The results of this study will inform priority setting and tailoring of our interventions to meet the unique needs of individuals and populations that our cancer prevention interventions serve.

To this end, our research questions are as follows: (1a) what is the impact of the first two waves of the COVID-19 pandemic (March 2020–February 2021) on four MRFs (i.e., tobacco use, physical activity, fruit and vegetable consumption, alcohol use) among people in Alberta compared to the pre-COVID-19 period (March 2019–February 2020) and (1b) do the impacts of the COVID-19 pandemic on four MRFs vary by socioeconomic status (SES), as measured by material deprivation?

## Methods

### Data source

To address these research questions, we used data from three cycles (2018–2021) of the Alberta Community Health Survey (ACHS), a cross-sectional telephone survey administered by Alberta Health Services to a random sample of people living in the province of Alberta who are ages 18 and older. The purpose of this survey is to monitor data on health behaviours, social determinants of health, and health outcomes. This cross-sectional study was designed to capture responses across Alberta, including surveying individuals living in rural and remote regions. Ethics approval was obtained from the University of Calgary’s Conjoint Health Research Ethics Board (#21-0538).

### Sampling and data collection

The ACHS is administered annually since 2014 and has a target sample size of approximately 4000 responses per cycle. The sampling frame is developed to have high-quality geographically representative estimates. The population of Alberta is sampled through random digit dialing, using geographic specificity, of both cell phone and landline numbers. As of 2017, a web-based survey was available to those who do not wish to complete the survey by phone. Data collection occurs for a full year starting in September.

### Survey weights

Sample weights generated by Alberta Health were applied to each survey to approximate the distribution of adults living in Alberta. Survey weights were estimated using iterative post-stratification methods that account for sampling design and telephone types. Weights were post-stratified based on month, age, gender, health regions, and response type. The sum of the weights was calibrated to the sample size of the cycle (Alberta Health, 2019).

### Outcome measures

Data were collected on four MRFs: tobacco use, physical activity, fruit and vegetable consumption, and alcohol use. Cut-offs were created based on whether respondents met the national or international guidelines for each risk factor. For fruit and vegetable consumption, we chose a cut-off of five servings of fruit and vegetables to align with the WHO recommendation for adults (World Health Organization, 2003). For physical activity, the Canadian Society of Exercise Physiology recommends that adults participate in 150 min of moderate-to-vigorous aerobic physical activity per week (Canadian Society of Exercise Physiology, 2011). For tobacco use, Health Canada’s Tobacco Strategy recommends quitting use of commercial tobacco products entirely (Health Canada, 2020); therefore, respondents were coded as meeting this guideline if they were not current or occasional tobacco smokers and did not use smokeless tobacco products. Finally, the Canadian Centre on Substance Use and Addiction provides guidelines for “low-risk” drinking to reduce the long-term health risks and short-term harms associated with alcohol consumption. Based on these guidelines, we generated a two-level variable indicating whether the respondent met the guideline for low-risk drinking (Butt et al., 2011).

### Exposure measure

For both research questions, a binary time period variable was generated based on the month the respondent participated in the survey and categorized as responding to the survey in the pre-pandemic period (March 2019 to February 2020) or during the pandemic (March 2020 to February 2021). The first case of presumptive COVID-19 in Alberta was reported on March 5, 2020 (Government of Alberta, 2021); therefore, in this study, we consider March 2020 to be the first month of the pandemic in Alberta.

### Covariates

Data on individual determinants such as gender, age, household composition, ethnicity, and geographic region were included in the ACHS. Finally, three variables (household education, home ownership, and food insecurity) were combined to create a measure of material deprivation, the Canadian Deprivation Index (CDI). Responses to a question on household education were categorized into 3 groups (not completed high school, completed high school/certificate, university degree or higher). Homeownership grouped respondents into a binary variable based on whether they were renters or homeowners. The food insecurity questions asked individuals whether they were worried about running out of food due to not having enough money and responses were grouped into 3 categories (often true, sometimes true, never true). Based on responses to these three questions, responses were given a total score ranging from 1 to 5 where 1 was the least deprived and 5 was the most deprived. Based on the distribution of this 5-level variable, a binary variable was created combining the top two least deprived groups and the bottom three most deprived groups. This variable is used as a measure of SES and has been used by the Government of Alberta for research purposes (Government of Alberta, 2015).

### Statistical analysis

To address the first research question, three cycles were pooled to create a dataset (*n*=11,249) covering one full year before the beginning of the COVID-19 pandemic in Alberta (March 2019–February 2020) and one full year during the pandemic (March 2020–February 2021). First, we conducted weighted and unweighted univariate analysis generating proportions and frequencies for the study variable. Second, we conducted bivariate analysis generating percent distributions and 95% confidence intervals for each study variable by time period (pre- vs. during pandemic).

Third, we conducted crude (unadjusted), age and gender adjusted, and fully adjusted logistic regression analyses to examine the odds of meeting recommended guidelines on four MRFs during the COVID-19 pandemic, compared to the year prior to the pandemic (control period). Potential confounders (age, gender, household composition, ethnicity, geographic region) are included in the fully adjusted logistic regression models. In all analyses, we interpreted two-tailed *p* values < 0.05 as statistically significant.

Finally, to assess whether the odds of meeting the recommended guidelines on four MRFs during the pandemic compared to prior to the pandemic varied by a measure of material deprivation, the CDI was assessed as an effect modifier on the relationship between time period and each MRF. CDI was found to be an effect modifier on the relationship between time period and physical activity and tobacco use (see Appendix Table 5); therefore, crude, age and gender adjusted, and fully adjusted logistic regression analyses were stratified by CDI and odds ratios and confidence intervals were generated for each level of CDI. All analyses were performed using SAS Studio version 9.4. Weights were applied to each survey to approximate the distribution of adults living in Alberta. Unweighted estimates are presented in the Appendix.

## Results

We included 11,249 survey participants from three cycles of the ACHS. The weighted percent distribution of study variables is presented in Table 1. We found statistically significant differences between the two time periods for gender, age, ethnicity, region, and CDI (Table 2). At both time periods, there were more female respondents than male respondents and this difference increased during the pandemic period. Respondents were more likely to be 65+ during the pandemic period and less likely to be ages 18–34, compared to the pre-pandemic period. Respondents were more likely to be visible minorities in the pandemic period and more likely to be living in Calgary or Edmonton zones, compared to the pre-pandemic period. Finally, respondents were less likely to have high material deprivation in the pandemic period compared to the pre-pandemic period. These differences in sample characteristics were likely the result of differences in respondents between the two time periods, rather than a change in population characteristics. These variables were included in the logistic regression analysis as potential confounder variables because of their independent association with the exposure variable (time period) and the outcome variables. In addition, we observed statistically significant differences in percent of individuals meeting recommended guidelines for tobacco use (*p*=0.0016), physical activity (*p*=0.0317), and fruit and vegetable consumption (*p*<0.0001) by time period.

**Table 1:** Weighted percent distribution of study variables (*n*=11,249)

	Percent (frequency)
Outcome variables	
Tobacco use	
Not a tobacco user	85.9 (8968)
Current or occasional user^a^	14.1 (1477)
Physical activity	
At least 150 min per week	64.3 (6580)
Less than 150 min per week^b^	35.7 (3656)
Fruit and vegetable consumption	
At least five servings per day	27.5 (2859)
Less than five servings per day^c^	72.5 (7533)
Alcohol use	
Meeting low-risk drinking guidelines	53.5 (5473)
Exceeding low-risk drinking guidelines^d^	46.6 (4766)
Exposure variables	
Time period	
March 2019–February 2020	52.7 (5515)
March 2020–February 2021	47.3 (4941)
Gender	
Male	47.3 (4871)
Female	52.7 (5418)
Age (years)	
18–34	23.4 (2421)
35–49	27.6 (2861)
50–64	28.1 (2908)
65+	20.9 (2163)
Household composition	
Living alone	14.3 (1489)
Living with adults	52.1 (5433)
Living with kids	33.6 (3501)
Ethnicity	
Non-visible minority	81.5 (8358)
Visible minority	18.5 (1897)
Alberta health zone	
Calgary region	38.6 (3929)
Edmonton region	30.9 (3142)
North	10.4 (1058)
Central	12.2 (1244)
South	7.9 (805)
Canadian Deprivation Index	
Low material deprivation	81.9 (8250)
High material deprivation	18.1 (1818)

^a^Current or occasional user of tobacco (including cigarettes, cigars, and non-smoking tobacco such as chew or snuff)

^b^Engages in, on a typical week, less than 150 min of vigorous or moderate physical activity per week

^c^Consumes less than 5 servings of fruits (including fruit juices) and vegetables per day

^d^Exceeds recommended guidelines for alcohol use which includes consuming more than 3 (for women) and 4 (for men) alcoholic drinks in one sitting, consuming more than 10 (for women) and 15 (for men) alcoholic drinks in one week, having no days in a week where they do not consume any alcoholic drinks, and consuming any alcoholic drinks while pregnant

**Table 2:** Weighted percent distribution of independent variables by time period (*n*=11,249)

	March 2019–February 2020	March 2020–February 2021	*p*-value
Tobacco use			0.0016
Not a tobacco user	84.8	87.0	
Current or occasional user	15.2	13.0	
Physical activity			0.0317
At least 150 min per week	65.2	63.2	
Less than 150 min per week	34.8	36.8	
Fruit and vegetable consumption			<0.0001
At least five servings per day	35.1	19.0	
Less than five servings per day	64.9	81.0	
Alcohol use			0.2274
Meeting low-risk drinking guidelines	52.9	54.1	
Exceeding low-risk drinking guidelines	47.1	45.9	
Gender			0.0018
Male	48.8	45.7	
Female	51.2	54.3	
Age (years)			<0.0001
18–34	24.9	21.7	
35–49	27.7	27.6	
50–64	28.0	28.2	
65+	19.4	22.5	
Household composition			0.1614
Living alone	13.9	14.8	
Living with adults	51.8	52.5	
Living with kids	34.3	32.8	
Ethnicity			0.0012
Non-visible minority	82.7	80.2	
Visible minority	17.3	19.8	
Alberta health zone			<0.0001
Calgary region	37.3	40.1	
Edmonton region	29.7	32.2	
North	11.6	9.1	
Central	12.9	11.5	
South	8.6	7.1	
Canadian Deprivation Index			<0.0001
Low material deprivation	80.3	83.8	
High material deprivation	19.7	16.2	

Table 3 presents results from the weighted logistic regression analysis assessing the odds of meeting recommended guidelines for four MRFs. Model 1 presents the crude odds of meeting recommended guidelines for four MRFs during the pandemic compared to the pre-pandemic period. Results from model 1 indicate that participants had slightly higher odds (OR: 1.20; 1.07–1.34) of reporting not being a current or occasional tobacco user during the pandemic compared to the pre-pandemic period. Participants had slightly lower odds (OR: 0.92; 0.84–0.99) of reporting getting at least 150 min of moderate or vigorous physical activity per week during the pandemic compared to the pre-pandemic period. In addition, participants had lower odds (OR: 0.43; 0.40–0.48) of meeting the fruit and vegetable guidelines during the pandemic period, compared to the pre-pandemic period.

**Table 3:** Weighted crude, sex and age adjusted, and fully adjusted models of the odds of meeting recommended guidelines for four modifiable risk factors during the COVID-19 pandemic compared to the pre-COVID-19 period

	Model 1: crude	Model 2: age and gender adjusted	Model 3: fully adjusted
Tobacco use			
Not a tobacco user	1.20* (1.07–1.34)	1.17** (1.04–1.31)	1.10 (0.98–1.24)
Physical activity			
At least 150 min per week	0.92* (0.84–0.99)	0.93 (0.85–1.01)	0.94 (0.86–1.02)
Fruit and vegetable consumption			
At least five servings per day	0.43*** (0.40–0.48)	0.42*** (0.38–0.46)	0.42*** (0.38–0.46)
Alcohol use			
Meeting low-risk drinking guidelines	1.05 (0.97–1.13)	0.99 (0.91–1.07)	0.95 (0.87–1.03)

* *p*<0.05; ** *p*<0.01; *** *p*<0.001

Results of the age and gender adjusted model (model 2) indicate that participants had slightly higher odds (OR: 1.17; 1.04–1.31) of reporting not being a current or occasional tobacco user during the pandemic compared to the pre-pandemic period. Participants had lower odds (OR: 0.42; 0.38–0.46) of meeting the fruit and vegetable guidelines during the pandemic period, compared to the pre-pandemic period, adjusting for age and gender.

Results from the fully adjusted model (model 3) indicate that participants had lower odds (OR: 0.42; 0.38–0.46) of meeting the fruit and vegetable guidelines during the pandemic period, compared to the pre-pandemic period. After adjusting for age, gender, household composition, ethnicity, and region, there were no observed statistically significant differences in odds of meeting recommended guidelines for tobacco use, physical activity, or alcohol use.

Table 4 presents the weighted fully adjusted logistic regression results for the odds of meeting recommended guidelines for four MRFs during the COVID-19 period compared to the pre-pandemic period, and these results are presented stratified by level of material deprivation (low material deprivation, high material deprivation). Participants experiencing high material deprivation have higher odds (OR: 1.36; 1.08–1.71) of not being a current or occasional tobacco user during the pandemic period compared to the pre-pandemic period, controlling for age, gender, household composition, ethnicity, and region. Participants experiencing high material deprivation have lower odds (OR: 0.65; 0.53–0.81) of reporting meeting recommended guidelines for physical activity in the pandemic period, compared to the pre-pandemic period, controlling for age, gender, household composition, ethnicity, and region. Finally, participants experiencing both low (OR: 0.40; 95% CI: 0.36–0.45) and high (OR: 0.50; 95% CI: 0.38–0.65) material deprivation report lower odds of meeting recommended guidelines for fruit and vegetable consumption during the pandemic period compared to the pre-pandemic period, controlling for age, gender, household composition, ethnicity, and region. There was no statistically significant difference in odds based on level of material deprivation for the relationship between time and meeting low-risk drinking guidelines.

**Table 4:** Weighted crude, fully adjusted models of the odds of meeting recommended guidelines for four modifiable risk factors during the COVID-19 pandemic compared to the pre-COVID-19 period, stratified by level of material deprivation

	Fully adjusted model^a^	
	Low material deprivation	High material deprivation
Tobacco use		
Not a tobacco user	0.97 (0.84–1.12)	1.36** (1.08–1.71)
Physical activity		
At least 150 min per week	0.99 (0.90–1.08)	0.65*** (0.53–0.81)
Fruit and vegetable consumption		
At least 5 servings per day	0.40*** (0.36–0.45)	0.50*** (0.38–0.65)
Alcohol use		
Meeting low-risk drinking guidelines	0.98 (0.89–1.08)	0.91 (0.74–1.13)

* *p*<0.05; ** *p*<0.01; *** *p*<0.001.

^a^Adjusted for age, gender, household composition, ethnicity, and region

## Discussion

In this study, we compared the differences in MRFs among adults in Alberta using repeated cross-sectional surveys administered one year prior to the COVID-19 pandemic and one year during the pandemic. We also examined the impact of the pandemic on four MRFs and whether this impact differs based on level of material deprivation. Below we describe our results in relation to the emerging evidence across the four MRFs.

### Tobacco use

Overall, we observed higher odds of reporting meeting recommended guidelines for tobacco use during the pandemic period compared to the pre-pandemic period when controlling for age and gender, but these odds were no longer statistically significant once we controlled for household composition, ethnicity, and region. The current relationship between the COVID-19 pandemic and patterns of tobacco use remains unclear. Some researchers who have observed decreases in tobacco use or increases in cessation attempts during the pandemic have hypothesized that the influx of information regarding the higher chances of getting COVID-19 for tobacco users, and worse outcomes if infected (Jackson et al., 2020; Vardavas & Nikitara, 2020), resulted in increased numbers of people quitting tobacco (Yang & Ma, 2021). Other researchers have hypothesized that increased time spent at home due to COVID-19 restrictions may result in individuals quitting smoking to avoid increased second-hand smoke exposure to household members (Jackson et al., 2021). In contrast, researchers have found increases in tobacco use among people who use tobacco, particularly associated with increased stress and anxiety because of the pandemic and its restrictions (Yingst et al., 2021).

We observed higher odds of not being a current or occasional tobacco user during the pandemic compared to the pre-pandemic period among people experiencing high material deprivation. We hypothesize that increased cessation support targeting individuals with lower SES could have contributed to the increase in individuals reporting not being current or occasional tobacco users during the first year of the pandemic. Within the context of Alberta and risks associated with COVID-19 transmission among tobacco users, the provincial government provided additional funding during the COVID-19 pandemic to smoking cessation mediations for people receiving a drug benefit supplement through income assistance (Alberta Health Services, n.d.).

### Physical activity

Our findings of decreasing physical activity during the COVID-19 pandemic have been consistently observed in studies examining this relationship (Di Sebastiano et al., 2020) and recent systematic and scoping reviews (Stockwell et al., 2021). We also found lower odds of reporting meeting recommended guidelines for physical activity during the pandemic period compared to the pre-pandemic period, but these were no longer statistically significant once our model was adjusted for confounding factors.

We observed lower odds of meeting recommended guidelines for physical activity during the pandemic compared to the pre-pandemic period among people experiencing high deprivation. Access to physical activities, fitness facilities, and team and individual sports was impacted by restrictions and subsequent closures of fitness facilities in Alberta in the middle of the second wave (late November 2020). As Albertans shifted their physical activity online and in their homes and neighbourhoods, factors such as home and yard size, neighbourhood safety, stable internet connections, and cost of fitness equipment (Dunton et al., 2020) may have contributed to the disproportionate impact of the COVID-19 pandemic on the physical activity of individuals experiencing high material deprivation observed in this study. Furthermore, there is emerging evidence that people who were overweight or obese during the pandemic were at greater risk of experiencing severe outcomes of COVID-19 (Sawadogo et al., 2022), which may have influenced individuals to increase their physical activity, if they had the resources to do so. This supports the need to examine changes in the health behaviours stratified by markers of SES, given that decreases in physical activity due to the pandemic have been widely reported (Meyer et al., 2020), but there remains a paucity of studies examining differences based on a marker of SES.

### Fruit and vegetable consumption

We also observed lower odds of meeting recommended guidelines for fruit and vegetable consumption during the pandemic compared to the pre-pandemic period. This aligns with multiple studies finding that fruit and vegetable intake has been negatively impacted by the pandemic (Bin Zarah et al., 2020). Several reasons for the decrease have been proposed — first, to avoid contact with others, individuals may reduce the number of times they visit grocery stores and therefore may opt for non-perishable items (Chenarides et al., 2021; Niles et al., 2020). Moreover, disruptions in supply chains have led to lower stock and increased costs of fruits and vegetables (Bakalis et al., 2020; Mead et al., 2020). Finally, some studies found individuals were less likely to buy fresh fruits and vegetables due to concerns over contamination (Niles et al., 2020).

Despite lower odds of reporting meeting fruit and vegetable guidelines during the pandemic compared to the pre-pandemic period, no differences were observed between people experiencing high and people experiencing low material deprivation. This contrasts with findings from Litton and Beavers (2021) which found that respondents in Michigan experiencing food insecurity were more likely to report decreasing their fruit and vegetable consumption since the beginning of the pandemic. This difference in findings could be attributed to differences in pandemic support programs offered in Canada and the United States. In Canada, the proliferation of the Canada Emergency Response Benefit (CERB), providing financial support to employed and self-employed Canadians affected by COVID-19, could have helped to supplement the lost wages and support the purchase of fruits and vegetables at the same rate as those who did not lose wages due to the pandemic. One simulation study conducted in 2020 found the lowest income households may benefit the most from CERB as it offers higher income than previous wages (MacGee et al., 2020). Overall, the certainty of evidence surrounding changing prevalence of food insecurity in Canada during the COVID-19 pandemic is still low (National Collaborating Centre for Methods and Tools, 2021) and more data are needed on the differences in health behaviour changes among different employment groups and among recipients of CERB to support this interpretation.

### Alcohol use

Our study did not find any statistically significant differences in meeting the low-risk drinking guidelines during the COVID-19 pandemic compared to the pre-pandemic period. This finding is supported by a Canada-wide poll conducted that found that approximately 12.8% of respondents in the prairies (i.e., Alberta, Saskatchewan, and Manitoba) stated that it had increased and 12.6% had stated that it had decreased (Canadian Centre on Substance Use and Addiction, 2020). In contrast, in Ontario, 18.3% of respondents reported their alcohol consumption had increased while only 12.7% reported it had decreased. This regional difference may be explained by differences in the timing of public health measures. In Alberta, restaurants and bars remained open, with restrictions, for much longer than those in other provinces. While liquor stores did remain open in all provinces during the COVID-19 pandemic, data from Ontario indicate that alcohol sales significantly increased during the pandemic, suggesting that individuals are drinking at home more instead of drinking in restaurants and bars (Zipursky et al., 2021). That being said, alcohol-related emergency department (ED) visits increased during the COVID-19 pandemic in Alberta (Rennert-May et al., 2021) but decreased in Ontario (Zipursky et al., 2021). This indicates that while population-level alcohol use in Alberta may not have statistically significantly increased, there may be significant increases in alcohol use among a subset of the population that might not have been captured by the outcome used to measure low-risk drinking in the present study. While our study did not find evidence of differences in changes in meeting the low-risk drinking guidelines due to the COVID-19 pandemic by material deprivation, some emerging research has found income-based disparities in ED visits due to alcohol (Myran et al., 2021), which may not be captured by the lower threshold of alcohol use needed for our outcome measure.

## Strengths and limitations

The use of one year of pre- and during pandemic data allows for comparisons made based on the unique experiences during the COVID-19 pandemic and not due to seasonal fluctuations in health behaviours. Additionally, the use of repeated annually collected cross-sectional design allowed for a breadth of data collected on health behaviour with standardized, valid, and reliable measures of MRFs.

The detailed questions included in this survey on MRFs allowed us to determine whether individuals were meeting national and international guidelines on four common health behaviours. Despite this strength, our survey did not include a measure on vaping tobacco products. We therefore anticipate that our findings are underestimating the prevalence of tobacco usage in Alberta, particularly among younger individuals. Recent studies have indicated higher proportions of e-cigarette use than cigarette use among Canadian youth (Chaiton et al., 2022). Moreover, the objective of this paper was to examine whether there were changes in meeting guidelines for 4 MRFs during the first year of the pandemic which is important for understanding the potential impact of the pandemic on future risk of chronic disease. Future research could explore more precise changes in health behaviours by examining these outcomes using continuous or ordinal measures.

Our cross-sectional research study was unable to examine changes over time in the MRFs due to the first two waves of the pandemic. Future longitudinal studies will be needed to further understand this relationship. Despite this, the relationships explored in this project are primarily exploratory and it is reasonable to investigate these questions using cross-sectional methods before conducting more cost-intensive longitudinal studies. Despite the large sample size of the present study, we did not have enough statistical power to disaggregate ethnicity beyond a crude two-level. Future studies should examine the health impacts of the pandemic based on this important social determinant of health.

The self-reported nature of the ACHS may introduce social desirability bias, in which respondents may over-estimate their healthy behaviours. Despite this concern, we anticipate that this bias may inflate the amount of people reporting meeting recommended guidelines for MRFs but that this inflation of responses would be uniform in both periods; therefore, any observed trends in the association between time and modifiable risk factors are likely to remain consistent.

The surveying for the ACHS is conducted annually from September to August. In order to test the impacts of the first year of the pandemic, we used a cut-point (i.e., March 2020) which did not align with the data collection time periods. This impacted the sample weights and therefore, the characteristics of participants in each time period differed by gender, age, ethnicity, and region; therefore, these findings may be susceptible to selection bias which decreases the representativeness of the dataset. We have presented the unweighted analysis in the Appendix for comparison and while some differences in statistical significance in the fully adjusted models exist due to wider confidence intervals in the unweighted analysis, the direction and magnitude of the relationships is similar between the weighted and unweighted analysis.

These variables were thus considered potential confounders and adjusted for in the models presented but we were only able to control for a small number of covariates which is not a comprehensive list of all potential confounders that might influence this relationship. Finally, research in context of the COVID-19 pandemic is ongoing; therefore, continued surveillance of this issue is needed across geographic locations. Moreover, our study only examines short-term effects and there may be a lag in pandemic effects on health behaviours; therefore, longitudinal survey methods are needed to assess this question more accurately.

## Conclusion

The COVID-19 pandemic’s social and economic shocks created profound disruptions in people’s daily lives and the health impacts of these disruptions have been the focus of a growing body of research. Yet, these studies often present a population-level view of the impact of the pandemic on MRFs which can mask the variation in impacts across, particularly disadvantaged, social groups. Therefore, it is important to interpret these findings within the context of social and economic inequities. The widely reported decreases in physical activity may only be concentrated within a subpopulation experiencing some form of deprivation, as observed in this study, which warrants a more targeted public health implementation approach. Interestingly, improvements in tobacco use were concentrated in the high material deprivation group, which indicates the success of increased funding to initiatives targeting individuals on income support or could indicate a renewed desire to quit using tobacco due to the messaging around the connection between tobacco use and COVID-19.

## Contributions to knowledge

What does this study add to existing knowledge?

- Using repeated cycles of a provincial population health survey, this study examines the impact of the first year of the pandemic on four important modifiable risk factors and examines the differences by level of material deprivation.
- This study found lower odds of meeting recommended guidelines for fruit and vegetable consumption at a population level during the pandemic, compared to the pre-pandemic period, but did not vary according to level of material deprivation.
- Individuals experiencing high material deprivation were less likely to meet physical activity guidelines and more likely to not be current tobacco users during the pandemic, compared to the pre-pandemic period.

What are the key implications for public health interventions, practice, or policy?

- When planning interventions for key MRFs in the COVID-19 recovery period, practitioners need to design interventions that are tailored to the unique experiences of those with high material deprivation to begin to address the inequitable impacts of the COVID-19 pandemic.
- Given the regional differences in public health actions taken by municipal and provincial governments, it is important to interpret COVID-19 findings in the context of the region from which the data were drawn prior to implementing interventions to address MRFs.
- There is a need to further engage with communities experiencing these inequitable impacts to further understand the local context to inform intervention design.

## Data Availability

The data belong to Alberta Health and are not publicly available.

## Appendix

**Table 5:** Results from type 3 tests for multiplicative interactions between CDI and time period on the odds of meeting recommended guidelines for 4 modifiable risk factors

	Wald chi-square	Type 3 test *P*-value
Tobacco use	4.922	0.0265
Physical activity	7.781	0.0053
Fruit and vegetable consumption	1.066	0.3018
Alcohol use	0.487	0.486

**Table 6:** Unweighted percent distribution of study variables (*n*=11,249)

	Percent (frequency)
Outcome variables	
Tobacco use	
Not a tobacco user	84.7 (9516)
Current or occasional use	15.3 (1718)
Physical activity	
At least 150 min per week	63.6 (6916)
Less than 150 min per week	36.5 (3966)
Fruit and vegetable consumption	
At least 5 servings per day	26.6 (2959)
Less than 5 servings per day	73.5 (8184)
Alcohol use	
Meeting low-risk drinking guidelines	58.9 (6418)
Exceeding low-risk drinking guidelines	41.1 (4485)
Exposure variables	
Time period	
March 2019–February 2020	56.6 (6369)
March 2020–February 2021	43.4 (4880)
Gender	
Male	39.1 (4299)
Female	61.0 (6709)
Age (years)	
18–34	14.1 (1561)
35–49	19.9 (2203)
50–64	31.2 (3448)
65+	34.8 (3842)
Household composition	
Living alone	20.2 (2265)
Living with adults	56.2 (6296)
Living with kids	23.6 (2639)
Ethnicity	
Non-visible minority	86.8 (9569)
Visible minority	13.2 (1450)
Alberta health zone	
Calgary region	30.7 (3299)
Edmonton region	21.2 (2282)
North	18.4 (1974)
Central	18.2 (1958)
South	11.5 (1237)
Canadian Deprivation Index	
Low material deprivation	80.2 (8646)
High material deprivation	19.8 (2133)

**Table 7:** Unweighted percent distribution of study variables by time period (*n*=11,249)

	March 2019–February 2020	March 2020–February 2021	*p*-value
Tobacco use			0.0585
Not a tobacco user	84.2	85.4	
Current or occasional user	15.9	14.6	
Physical activity			0.0074
At least 150 min per week	64.6	62.2	
Less than 150 min per week	35.4	37.9	
Fruit and vegetable consumption			<0.0001
At least five servings per day	32.9	18.3	
Less than five servings per day	67.1	81.7	
Alcohol use			0.6574
Meeting low-risk drinking guidelines	59.1	58.6	
Exceeding low-risk drinking guidelines	41.0	41.4	
Gender			0.1595
Male	38.5	39.8	
Female	61.5	60.2	
Age (years)			<0.0001
18–34	12.0	16.9	
35–49	19.0	21.1	
50–64	32.0	30.1	
65+	37.0	31.9	
Household composition			0.0011
Living alone	21.2	19.0	
Living with adults	56.4	56.0	
Living with kids	22.5	25.0	
Ethnicity			<0.0001
Non-visible minority	89.6	83.4	
Visible minority	10.4	16.6	
Alberta health zone			<0.0001
Calgary region	27.2	35.3	
Edmonton region	18.9	24.4	
North	22.7	12.6	
Central	18.4	18.0	
South	12.8	9.8	
Canadian Deprivation Index			0.3759
Low material deprivation	79.9	80.6	
High material deprivation	20.1	19.4	

**Table 8:** Unweighted crude, sex and age adjusted, and fully adjusted models of the relationship between time period and the odds of meeting recommended guidelines for 4 modifiable risk factors

	Model 1: crude	Model 2: age and sex adjusted	Model 3: fully adjusted
Tobacco use			
Not a tobacco user	1.11 (1.00–1.23)	1.14* (1.03–1.27)	1.03 (0.92–1.16)
Physical activity			
At least 150 min per week	0.90 (0.83–0.97)	0.88** (0.82–0.96)	0.89** (0.82–0.97)
Fruit and vegetable consumption			
At least 5 servings per day	0.46*** (0.42–0.50)	0.45*** (0.41–0.49)	0.45*** (0.41–0.50)
Alcohol use			
Meeting low-risk drinking guidelines	0.98 (0.91–1.06)	1.06 (0.98–1.15)	1.04 (0.95–1.13)

* *p*<0.05; ** *p*<0.01; *** *p*<0.001

**Table 9:** Unweighted crude, fully adjusted models of the relationship between time period and the odds of meeting recommended guidelines for 4 modifiable risk factors stratified by level of material deprivation

	Fully adjusted model^a^	
	Low material deprivation	High material deprivation
Tobacco use		
Not a tobacco user	1.05 (0.91–1.21)	1.04 (0.84–1.29)
Physical activity		
At least 150 min per week	0.89* (0.81–0.98)	0.86 (0.71–1.04)
Fruit and vegetable consumption		
At least 5 servings per day	0.44*** (0.40–0.49)	0.44*** (0.34–0.57)
Alcohol use		
Meeting low-risk drinking guidelines	1.04 (0.95–1.15)	1.00 (0.81–1.22)

* *p*<0.05; ** *p*<0.01; *** *p*<0.001

^a^Adjusted for age, gender, household composition, ethnicity, and region

## Code availability

The analysis code is not available for this project.
